# Preschool mother-child emotional preparation program leads to significant improvement in autonomic regulation: a randomized controlled trial

**DOI:** 10.3389/frcha.2024.1308210

**Published:** 2024-04-29

**Authors:** Martha G. Welch, Robert J. Ludwig, Justin Kong, Virginia Rauh, Amie A. Hane, Marc Jaffe, Anna Witkowski, Michael M. Myers

**Affiliations:** ^1^ Department of Psychiatry, Columbia University College of Physicians & Surgeons, New York, NY, United States; ^2^ Department of Pediatrics, Columbia University College of Physicians & Surgeons, New York, NY, United States; ^3^ Department of Pathology & Cell Biology, Columbia University College of Physicians & Surgeons, New York, NY, United States; ^4^ Heilbrunn Department of Population and Family Health, School of Public Health, New York, NY, United States; ^5^ Department of Psychology, Williams College, Williamstown, MA, United States; ^6^ Children’s Learning Centers of Fairfield County, Stamford, CT, United States

**Keywords:** autonomic regulation, mother-child, socioemotional behavior, preschool behavior, emotional connection, autonomic conditioning, stress responding

## Abstract

**Introduction:**

Many studies have documented the profound impact that the mother-child relationship has on child sociality and behavior. However, the biological mechanisms that govern the relationship are poorly understood. We developed a mother-child emotional preparation program (MCEP), based on a novel autonomic nervous system learning mechanism. MCEP is hypothesized to condition the child's autonomic nervous system to better meet the preschool socioemotional classroom challenges.

**Methods:**

We conducted a randomized controlled trial of MCEP, comparing a group of children receiving standard curriculum with children receiving standard curriculum plus MCEP. Previously, we reported that the MCEP mother-child dyads (vs controls) were more emotionally connected at six months post intervention and MCEP children (vs controls) displayed better socioemotional behavior at home and in the classroom. At six months post intervention, mothers and children underwent a stressful interaction-interruption paradigm, during which we acquired child ECG. We analyzed heart rate and several measures of child heart rate variability obtained during the 10-minute post-stress recovery period.

**Results:**

We found that MCEP children showed better autonomic regulation following the stressor, as measured by lower heart rate (*p* = 0.017) and increased high frequency respiratory sinus arrhythmia (RSA) or vagal tone (*p* = 0.043). Surprisingly, despite a sample size limited by COVID (*n* = 12 and 12), the effect sizes were large (g's ranging from 0.89 to 1.09). In addition, we found significant correlations with large effect sizes between autonomic measures and scores on the Welch Emotional Connection Screen (WECS).

**Discussion:**

These findings support the conclusion that MCEP had a significant positive impact on child autonomic regulation in response to stress, which correlates with behavioral assessments of emotional connection. We discuss theoretical considerations and the implications of our findings for preschool education programs in general. This trial was retrospectively registered (clinicaltrial.gov registry NCT02970565) three months after the start of the first recruitment on April 9, 2019.

## Introduction

Antisocial behavior in preschool-aged children has been increasing for decades. In the US preschoolers now have the highest rates of school expulsion of all age groups (1). Antisocial behavior in childhood often leads to lifelong social exclusion and considerable personal distress and dysfunction into adulthood (2) and imposes high public and private expenditure for treatments (3, 4).

Antisocial behavior among preschool-aged children has worsened because of the COVID-19 pandemic and has been declared a *national emergency* (5).

Over the last 10 years, numerous reviews and meta-analyses have examined the efficacy and effectiveness of various intervention programs designed to address the rising problem of emotional, behavioral, and developmental disorders in preschool aged children, including psychosocial interventions for ADHD (6); psychological interventions targeting behavioral inhibition and anxiety (7); cognitive behavioral therapy (CBT) (8); school-based interventions to prevent anxiety and depression in young children (9); Incredible Years Teacher Classroom Management (IYTCM) for adverse socioemotional behavior (10), and Parent management training (PMT) for behavior problems (11).

Overall, results of such interventions show limited or inconclusive effect on overall adverse classroom behavior and most are not suitable in their present form for scaling, due to small effect size and/or the limited availability, length and cost of treatment programs. The situation has left teachers increasingly burned out, and preschool educators struggling to find alternative solutions.

To address the preschool behavioral problem (12), we co-developed a novel Emotional Preparation program (MCEP) with Children's Learning Centers of Fairfield County (CLC), a leading community-based pre-school education program serving ∼1,000 families annually in Stamford, CT. MCEP is a preventative group family intervention facilitated by trained specialists and designed to help parents prepare their child for the pre-school socioemotional experience. We conducted a randomized controlled trial (RCT) of MCEP at CLC (ClinicalTrials.gov Identifier: NCT03442439), comparing a group of children receiving CLC's standard curriculum with a group receiving standard curriculum plus MCEP.

Previously, we reported that the MCEP mother-child dyads (vs. controls) were more emotionally connected at six months post accrual and MCEP children (vs. controls) displayed better socioemotional behavior at home and in the classroom (13). In addition to behavioral benefits, we recorded real-time physiological data to better inform decisions on whether to integrate the preventative practices into CLC's preschool standard curriculum. Specifically, we examine here the effects of MCEP on child physiological responding to stress, as measured by heart rate and heart rate variability in-vivo, during a 20-min recovery period after a series of interrupted interactions with their mother.

It is well-established that individual differences in the quality of parent-child interaction are associated with infant and child stress response at a physiological level (14, 15). Electrocardiogram (ECG) methodology has been utilized by previous studies to measure *in vivo* cardiac activity as an assessment of stress reactivity and recovery (16). Infant parasympathetic regulation during a relational stressor is associated with maternal sensitivity (17, 18), parental responsiveness (19), and mother-child coordination of affective behaviors (20).

We have proposed that mothers and infants are biologically designed to co-regulate one another’s autonomic state through an autonomic ‘calming cycle’ conditioning process. The process starts during gestation and results in the formation of an interpersonal autonomic socioemotional reflex (ASR) (21, 22), which can be measured by Welch Emotional Connection Screen (WECS) (23).

Measuring the mother and infant relationship in terms of the autonomic socioemotional reflex (ASR) requires rethinking the biological mechanisms mediating the mother-child relationship. Conventional constructs, such as attachment and bonding, focus on conscious and unconscious cortical learning mechanisms. In contrast, ASR theory posits that mother/infant emotions are controlled by highly conserved primitive learning mechanisms operating outside of consciousness. ASR theory proposes that specialized primary autonomic (i.e., cardiac) reflexes form between mother and fetus during gestation via autonomic learning or conditioning.

We posit that the ASR (24) is present in all vertebrate species, and is arguably mediated by the oldest and most highly conserved learning mechanism—*functional Pavlovian or autonomic conditioning* (21, 25). Our theoretical advance is that the autonomic conditioning mechanism can be exploited (e.g., via calming cycle intervention) to lower average resting HR in the face of socioemotional challenge (26).

The idea was inspired by a phenomenon originally reported by Pavlov in 1925 when he described how the emotional relationship between a dog and trusted master profoundly impacted the dog’s cardiac function and behavior. Pavlov’s term for the phenomenon was “cardiac” or ’social’ reflex (27). We have applied Pavlov’s concept to the specific mother-child relationship. Due to their critical role in infant and child development, we have termed the mechanism the *autonomic socioemotional reflex (ASR)*.

The ASR is a special case of the highly conserved *orienting reflex.* Dysfunctional orienting is highly correlated with socioemotional pathologies in infants and children, including social fear, anger, anxiety, depression and autism. Orienting stems from activation of highly conserved autonomic defensive and appetitive motivational systems that evolved to sustain life, assuring the survival of species. In this respect, the ASR orienting phenomenon in humans does not differ significantly from other species, from which it was conserved.

The ASR mechanism provides a biological explanation for mother-infant behaviors that are measured on the uWECS. In previous studies, we have shown the orienting behaviors of preterm infants at 4 months as measured on the WECS correlated with cardiac physiology (Hane).

Theoretically, WECS behaviors reflect the autonomic physiology that is driving the behavior. Therefore, the WECS can be used to monitor the health of the mother/infant autonomic relationship. We have reported mother-child behavior as measured with the WECS In a separate report in this special issue (Welch et al). This study reports the cardiac.

Over the past few decades, vagal tone research among newborns and children has yielded important insights into social behavior, social interactions, and human psychology (28, 29). Heart rate variability, defined as the variation in intervals between consecutive heartbeats, has been used as a physiological indicator of regulatory processes, reflecting changes in autonomic regulation of heart rate. The sympathetic nervous system and parasympathetic nervous system both play a role in the regulation of heart rate, and to some extent can be indexed by measures of variability (30, 31). For example, the standard deviation of R-R peak intervals reflects overall variability, which is influenced by both sympathetic and parasympathetic nervous system activity (SNS and PNS respectively). Whereas, variation in successive R-R intervals, often termed high frequency HRV or respiratory sinus arrhythmia (RSA), largely reflects PNS activity mediated by the vagus nerve.

The theory that RSA (changes in heart rate with respiration} were due to variation in parasympathetic control of heart rate was originally based on Hering’s observations that specific vagal fibers that were cardioinhibitory had a respiratory rhythm (32). Confirmation from years of basic and clinical research by many investigators has led to the conclusion that measuring changes in heart rate that occur at frequencies associated with respiration provides an indirect index of vagal tone (33–35).

Vagal tone helps maintain the dynamic autonomic regulation important for cardiovascular health. In a healthy human heart, there is a dynamic relationship between the PNS and SNS. PNS control predominates at rest, resulting in an average (adult) HR of 75 bpm (36). The vagus nerve can exert its effects more rapidly (<1 s) than sympathetic nerves (>5 s) (37). Since these divisions can produce opposite actions on HR, their net effect on HR depends on their balance of activity. While the SNS often changes reciprocally to PNS activity, under some conditions the two can be activated or increased at the same time (i.e., co-activated) (38).

Our primary hypothesis in this study was that MCEP would lead to an increase in cardiac parasympathetic activity, indexed by the amplitude of RSA, or vagal tone. This was based on findings from our prior studies of the effects of Family Nurture Intervention (FNI) for preterm infants, an intervention with the same emotional connection goals as MCEP. In that trial, we found that FNI accelerated the maturation of vagal tone during the stay in the neonatal intensive care unit (Insert ref 46). In long term follow-up studies, FNI increased vagal tone out to 5 years of age (39). Accordingly, in the current trial of MCEP we hypothesized that the MCEP intervention, also designed to increase mother/child emotional connection would lead to increases in vagal tone.

Two secondary hypotheses were also specified based on prior results. As noted above we have recently published results from the preschool CLC RCT which showed that children randomized to the MCEP intervention group were more likely to be emotionally connected as indexed by WECS scores six months after the last intervention session (13). Here we tested the hypothesis that there would be significant relationships between emotional connection scores on the WECS and physiology. Also based on this prior paper we hypothesized that HR and/or vagal tone would be correlated with scores on the SWYC.

Hypothesis 1. At six months post intervention, children in the MCEP group (vs. control group) would have higher vagal tone and lower HR during the 20-min recovery period.

Hypothesis 2. Vagal tone and HR would be correlated (positively and negatively respectively) with scores on the WECS (i.e., higher WECS scores would be associated with higher vagal tone and lower HR during the 20-min recovery period.

Hypothesis 3. HR and vagal tone of children in the MCEP group (vs. control group) would be associated with better behavioral outcomes as assessed by the SWYC.

## Materials and methods

### Study design

This study was a parallel-group, single blind randomized controlled trial. The method is presented as per the CONSORT guidelines (40). This trial was registered in the clinicaltrial.gov registry (NCT03908268) on April 9, 2019 (Note that after registration the name of the intervention was changed from Family Nurture Intervention to Mother Child Emotional Preparation- MCEP). The central hypothesis of the RCT was that children in the treatment group would show increased short- and long- term emotional connection, as measured by the Welch Emotional Connection Screen (WECS). The study also assessed the impact of MCEP on child behavior in the home and classroom and autonomic markers of child emotion regulation. CLC's goal was to determine whether MCEP could be and should be added to CLC's standard curriculum.

### Participants and setting

MCEP was developed to be integrated into the standard curriculum at the CLC school locations. Children's Learning Center of Fairfield County (CLC), a community-based preschool facility that provides high quality, early childhood education programs for children between six weeks and five years of age at eight locations throughout Stamford, Connecticut (Children's Learning Centers of Fairfield County, 2016). Programs provided by CLC include Head Start, Early Head Start, and School Readiness and Child Development. Participants of the study were recruited by study staff at CLC orientations and teacher/parent meetings. Flyers were also distributed to CLC families during child drop-off and pick-up times. Teachers signed a consent form to enable collection of teacher-report data. CLC staff also discussed the study with enrolling parents and encouraged teachers to refer their students.

The National Education Association has placed a priority on socio-emotional learning for students and educators (41). Accordingly, CLC standard curriculum includes RULER, an evidence-based approach that teaches a child to recognize and labeling emotional feelings (42). The MCEP intervention compliments RULER but differs in strategy. MCEP engages the parents in emotional connection activities to be practiced in the home that proactively promote parent-child co-regulation, which in turn sustains positive feelings and ability to co-regulate with the teachers during the school day.

### Eligibility

Families were eligible for this study according to the following criteria: had a child that was 2–4.5 years of age at the recruitment date; mother was at least 18 years old; mother was able to speak, read, and write in English or Spanish; mother lived with her child full-time. In addition, the child had to be a singleton without a genetic or congenital disorder or motor disabilities. Mothers were excluded from the study if they had severe mental illness or any other medical conditions preventing play activities; were involved with the Department of Children and Families; struggled with drug or alcohol abuse; were advanced in pregnancy (2nd trimester or further) which would interfere with their ability to conduct lap-based procedures (described below); or were unable to commit to the study schedule.

### Consent procedures

Mothers were verbally consented and then asked to fill out Study Eligibility and Demographics Forms, as well as a CLC Release of Information Form. If an eligible mother did not have time, forms were completed over the phone later, and the CLC Release was signed at the first in-person contact at the time of their baseline assessment.

### Randomization process

**Figure 1:**
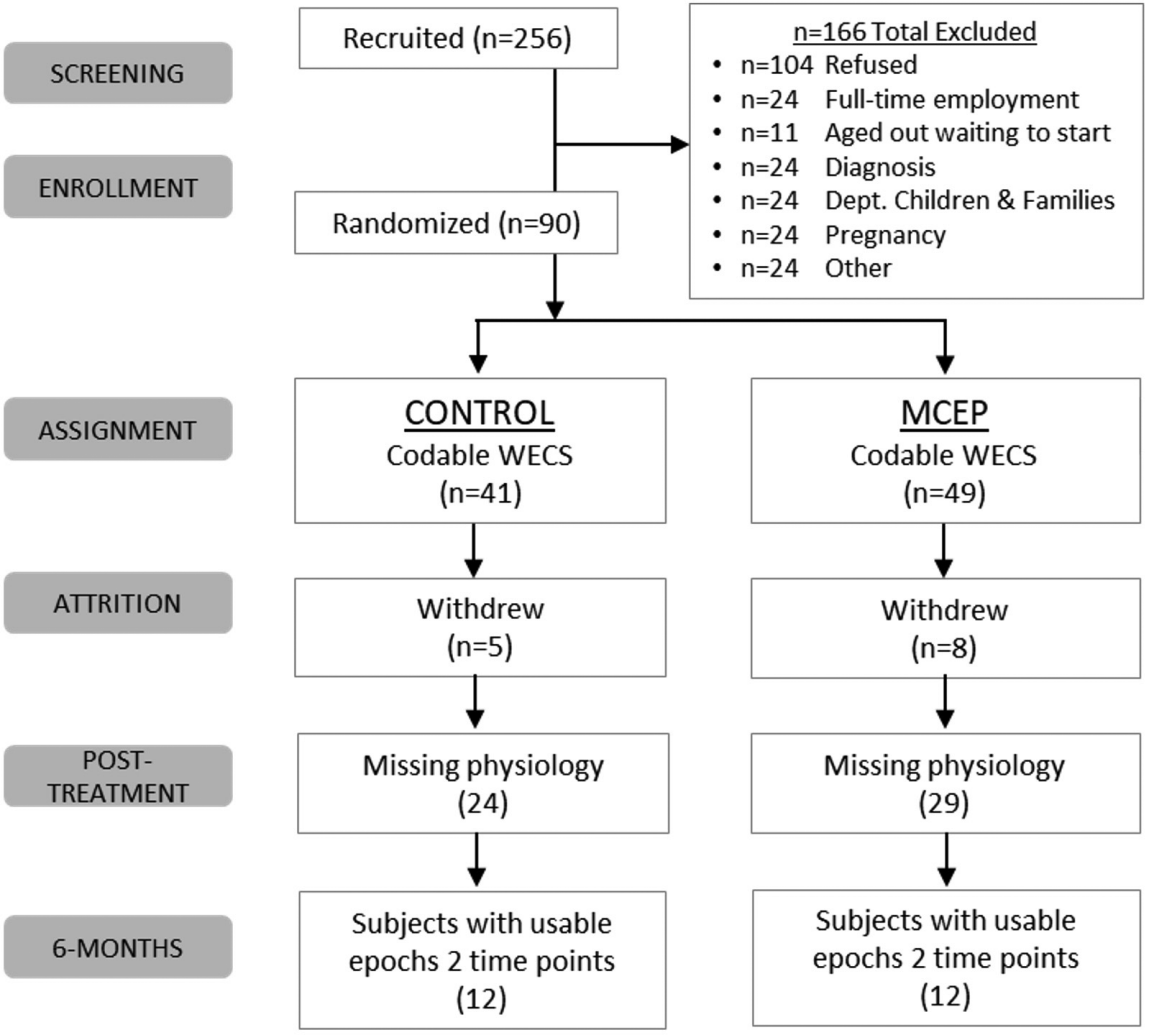
Consort chart. MCEP, Mother-Child Emotional Preparation; *n* = sample size; WECS, Welch Emotional Connection Screen.

Following consent, subjects were assigned to either one of two groups: An intervention group, which participated in the standard curriculum plus two to eight 2-hour calming sessions in a group setting; or, a control group, which received only the standard CLC curriculum (See Figure 1 Consort Chart).

The control group children participated in the standard CLC curriculum, with no additional procedures. Classroom activities varied by age and ability. Classroom structure also varied by program. For example, although most CLC students are enrolled in a full-day program, some attend a half-day program.

### Intervention methods

The intervention group dyads participated in MCEP sessions led by two specially trained licensed clinical social workers (Nurture Specialists). Two to eight dyads participated in each session, which was held in CLC space. In this study, the Nurture Specialists were licensed social workers. Sessions took place on a variable schedule over the course of sixteen weeks (i.e., mothers chose any 8 of 16 weeks to attend). The Nurture Specialists engaged mother-child dyads in calming sessions.

Calming sessions began with the child sitting facing mother on her lap. Behavior typically cycled through four distinct phases (43): (1) *Separate mother and child distress*. Mothers were asked to look at the child, describe behavior she wants to see from the child, and express any feelings in response to the child's current or home behavior. This typically elicited protest, crying or avoidant orienting behavior from the child. The child often pushed the mother away or tried to escape from the lap. (2) *Mutual engagement of distress*. While expressing her feelings, mothers would also express frustration and describe negative emotions, and sometimes cry. The mother's release of emotion, typically triggered a change of orientation from avoidant to attraction to the mother*.* (3) *Mutual resolution of distress*. At this point in the session, the child often responded to the mothers’ distress with sustained eye contact, tender behaviors and vocal communication, which moved the cycle in to the final stage. To aid in completing the cycle, Nurture Specialists encouraged the mother to use comfort touch, genuine emotional expression and eye contact. The mother's genuine expression of emotional feeling most often elicited reciprocal empathic response from the child. (4) *Mutual resolution and calm*. Mutual calm was observable as quiet embrace, eye contact and soothing talk. During this resolution phase, the dyad engaged in mutual comforting and settled into a state of mutual calm (i.e., autonomically co-regulated state), characterized by mother and child breathing calmly, maintaining a deep mutual gaze, and having open verbal and non-verbal communication with relaxation and reciprocal pleasure in each other's closeness.

Following the calming session, mothers were instructed to continue these calming cycles on a regular basis at home, especially when either child or mother was upset.

### Physiology study cohort

Among the 90 participants recruited for the initial emotional connection study from this RCT (44), largely due to the onset of the Covid pandemic, only 34 subjects were able to returned for ECG data collection at both the baseline and 6-month timepoints. Of these 34, 32 were able to provide epochs for HRV analyses at the baseline timepoint while 29 were able to provide epochs for HRV analyses at the 6-month timepoint. After filtering out all epochs that had a “Percent Good” value of less than 70%, we were left with 273 epochs from the baseline timepoint (provided by 31 participants) and 153 epochs from the 6-month timepoint (provided by 25 participants).

Of the remaining participants, 24 (*n* = 12 control and *n* = 12 MCEP) had both baseline and 6- month data to be used for statistical analyses (Figure 1). Demographic data (age, sex, etc.) of the 24 participants included in the statistical analysis are presented in Table 1.

**Table 1:** Demographics of subjects.

	SC (*n* = 12)	E-Prep (*n* = 12)
	Mean (SD)	Mean (SD)
Age at enrollment (years)	3.99 (0.48)	3.68 (0.21)
Age at 6-month follow-up (years)	4.54 (0.47)	4.23 (0.22)
Household size	4.17 (0.94)	4.42 (0.90)
	*N* (%)	*N* (%)
Parental marital status		
Married or living together	8 (66.7)	9 (75)
Hispanic ethnicity		
Mother	9 (75)	10 (83.3)
Father	9 (75)	11 (91.7)
Mother's education		
Some schooling	1 (8.3)	2 (16.7)
High school or GED	4 (33.3)	2 (16.7)
Some college or associates	5 (41.7)	7 (58.3)
Bachelors or graduate degree	2 (16.7)	1 (8.3)
Unknown	0 (0)	0 (0)
Father's education		
Some schooling	3 (25)	5 (41.7)
High school or GED	4 (33.3)	2 (16.7)
Some college or associates	4 (33.3)	2 (16.7)
Bachelors or graduate degree	0 (0)	2 (16.7)
Unknown	1 (8.3)	1 (8.3)
Employment status		
Mother is employed	5 (41.7)	5 (41.7)
Missing	0 (0)	1 (8.3)
State/federal assistance		
Yes	7 (58.3)	7 (58.3)
Unknown	2 (16.7)	0 (0)
Male	6 (50)	4 (33.3)

### Physiological assessment

**Figure 2:**
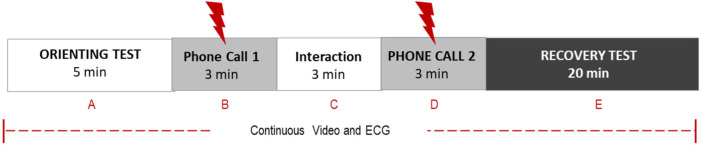
Parent-child stress recovery paradigm. This ∼30-min face-to-face communication paradigm is designed to determine the extent to which a parent-child dyad can return to baseline behavior (segment A) and parasympathetic/cardiac physiology following the dysregulation caused by two separation phone calls. Dyadic behaviors are coded from video recordings with Welch Emotional Connection Screen (WECS). Autonomic physiology is measured by heart rate and heart rate variability.

Electrocardiogram (ECG) data was collected and digitized at 1,000 Hz using a Noldus/Biopac MP160 System at approximately six months post-accrual, as part of the Parent-Child Stress Recovery Paradigm designed by authors Welch and Hane (Figure 2). For this study, we analyzed ECG data collected during the 20-minute recovery phase of the 30-min paradigm. The paradigm is designed to test the extent to which mother and child can recover (i.e., return to baseline parasympathetic/cardiac physiology) following a stressful (dysregulating) double separation. In the paradigm, during the 20-minute recovery period, the mother sat in a chair with the child facing her in her lap. There were no toys or distractions in the room. The mother was told to engage her child in conversation, and that she would receive two separate important three-minute phone calls during the interaction session. She was told that she should devote all her attention to these important calls and none to her child. Following each call, the mother was instructed to return her full attention to her child and resume their interaction.

### Welch emotional connection screen

Prior to the phone call disruption and during the 6-month post-enrollment follow-up, mothers and children were observed interacting in a 5 min face-to-face interaction that was later coded for EC with the WECS (23). The WECS is a brief observational tool that assesses the degree of emotional connection between mother and infants/children ages 0–6 years. Dyads are rated on a 9-point scale with 25 increments for: *Mutual Attraction* (use of shared gaze, proximality and touch); *Mutual Vocal Affect* (mutual use of voice); *Mutual Facial Expressiveness* (mutual facial affect); and *Mutual Sensitivity and Reciprocity* (mutual sensing and responding to each other) to initiate and sustain a connection with each other. A higher score indicates a dyad who is high on EC, manifesting the capacity to remain close, share affect, and engage in face-to-face interactions in a calm, mutually comfortable state of togetherness.

The WECS has been validated in preterm (23); full-term (45) infants and preschool-aged children (13).

### Heart rate variability analyses (vagal tone)

There are many analytical approaches to measuring RSA but all are based on capturing the amount of variation in heart rate that is associated with respiration. The first step in these analyses is locate the time when each non-artifact R-wave occurred. For this current study we used *R-DECO*, an open-sourced Matlab-based graphical user interface (46, 47) to automatically locate ECG R-wave peaks. Then, using the interactive graphic displays of R-DECO we inspected the ECG wave-forms and R-wave peak marks and manually inserted, deleted or moved peaks when appropriate.

Before computing various measures of RR-interval variability, using our own software (see definitions below), we detected and deleted RR interval values that were outside normal minimum and maximum RR- intervals. For these thresholds we used published data for three age ranges corresponding to the ages of children in our study (29, 47). These ranges are given in Table 2. In addition, we accepted RR-intervals only if successive R-R intervals changed by no more than 20%.

**Table 2:** R-R intervals for three age ranges.

Child	R-R interval		
Age range	Frequency range	Min ms	Max ms
2.5–3.49 Years	LF (Low)	428	800
3.5–4.49 Years	MF (Medium)	444	867
4.5–5.49 Years	HF (High)	460	923

Each 20-minute RR-interval file was analyzed in 3 min epochs with a sliding window which overlapped epochs by 1.5 min. Within each epoch, we calculated the percent of good data, that is, the total number of RR-intervals accepted divided by the total number of RR-intervals prior to the deletions based on the criteria described above. To increase data quality, statistical analyses were restricted to include only epochs that had percents good data greater than 70%. The values for a given subject included in these analyses were the medians from the included epochs. Subjects were only included in the analyses if they provided median values at both the baseline timepoint and 6-month timepoint. Overall, these procedures yielded an average of 8.8 good data epochs at baseline and 6.0 good epochs at the six-month follow-up.

Our software computed many parameters which followed the definitions and terms used by Shaffer and Ginsberg (Shaffer & Ginsberg, 2017).

*Heart Rate:* The inverse of an average of all good intervals within each epoch was used to compute mean heart rate (HR).

*SDNN (Standard deviation of normal-to-normal RR-intervals, a* time-domain measure of the standard deviation of R-R Intervals within an epoch, reflecting both parasympathetic and sympathetic influences on HR.

*RMSSD (Root mean square of successive differences), a* time-domain measure of differences between R-R intervals)*.* RMSSD is obtained by calculating each successive R-R interval between heartbeats and calculating the average change in R-R-intervals from one beat to the next. Then each of these values are squared and the result is averaged before the square of the total is obtained. Compared to SDNN, the RMSSD is thought to be affected more by parasympathetic activity.

*Frequency-Domain Measures of R-R Interval Variability.* Autoregressive modeling was used to separate heart rate variability into its component Low (LF) and High (HF) rhythms that operate in different frequency ranges. Spectral power in the LF and HF frequency bands was obtained by calculating the area under the R-R interval time series within each band’s frequency range: LF (0,040,15 Hz), HF (0.24–1.04 Hz). LF and HF values were log transformed (ln) prior to performing analyses.

*Respiratory Sinus Arrythmia (RSA)*. RSA was taken as the primary marker of cardiac-linked parasympathetic regulation. High resting RSA can represent a flexible and adaptive physiological response system to a challenge while a low resting RSA can reflect maladaptive regulatory mechanisms (48). RSA in this study was calculated using the Porges-Bohrer Method (Porges & Bohrer, 1990).

### Statistical analyses

Hypothesis 1. At six months post intervention, children in the MCEP group (vs. control group) would have higher vagal tone and lower HR during the 20-min recovery period.

This hypothesis was tested by comparing HRV value for MCEP subjects vs. controls at the six-month time point. However, the tests used (ANCOVAs) took into account the values for these parameters at baseline and, based on prior literature, also included age and sex as additional covariates. Following these analyses we computed effect sizes Hedge’s g (31), which essentially describes how large the mean effect of the intervention was compared the variance in the data.

Hypothesis 2. Vagal tone and HR would be correlated (positively and negatively respectively) with scores on the WECS (i.e., higher WECS scores would be associated with higher vagal tone and lower HR during the 20-min recovery period.

This hypothesis was tested by computing Pearson Product Moment correlations between WECS scores obtained during 5 min of face-to-face interactions while the child was on the mother’s lap at the 6-month time point and the HR and vagal tone values obtained during the 20-minute period of HRV analyses.

Hypothesis 3. HR and vagal tone of children in the MCEP group (vs. control group) would be associated with better behavioral outcomes as assessed by the SWYC.

This hypothesis was tested by computing Pearson Product Moment correlations between SYWC scores at the 6-month time point and the HR and vagal tone values obtained during the 20-minute period of HRV analyses.

## Results

Hypothesis 1. At six months post intervention, children in the MCEP group (vs. control group) would have higher vagal tone and lower HR during the 20-min recovery period.

**Figure 3:**
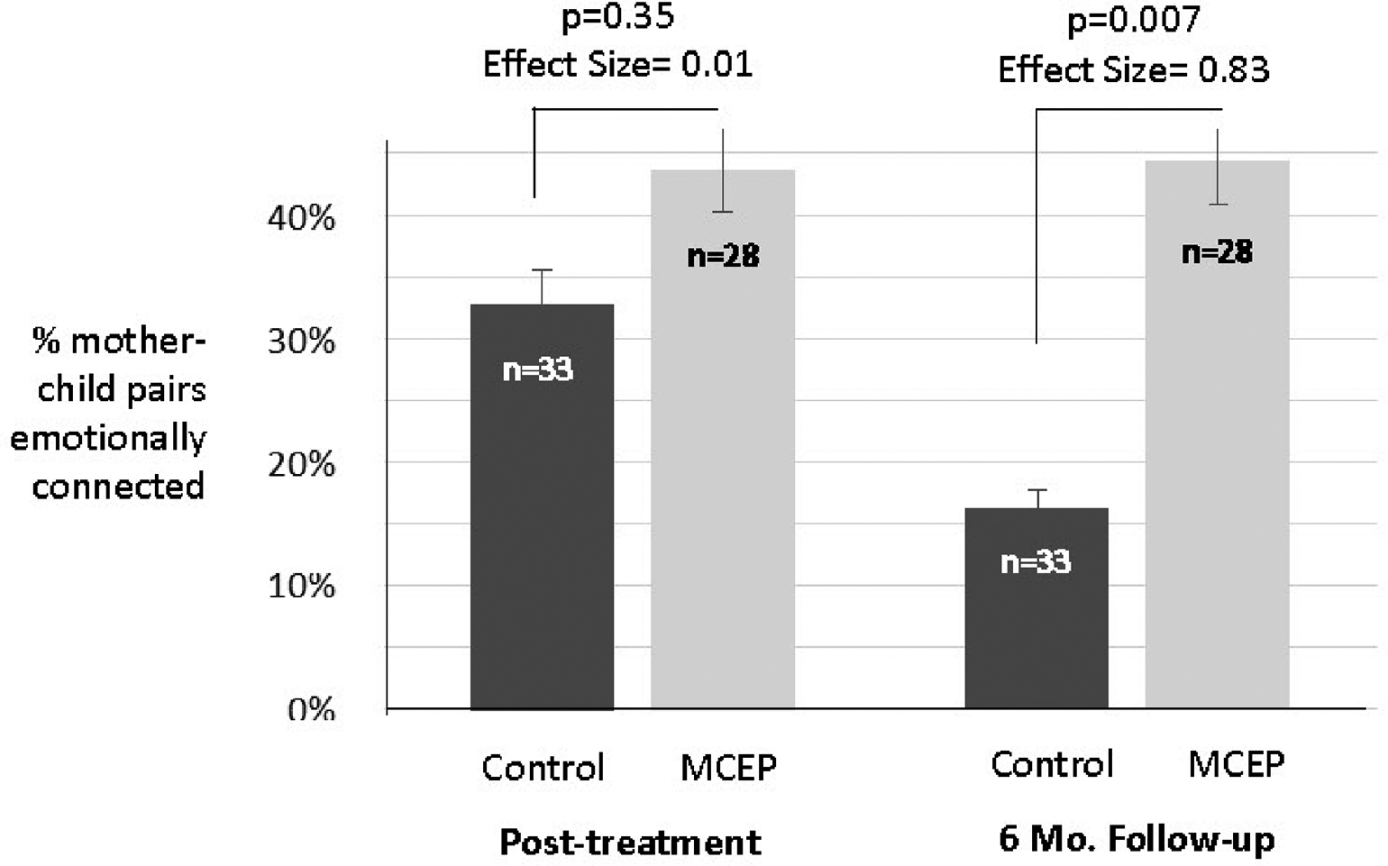
Heart rate and respiratory sinus arrythmia at 4 months. Graphs showing means (±SE) for HR (**A**) and RSA (**B**) for SC and MCEP children approximately 4 months after their last intervention session. Statistics were based on analyses of covariance with sex, age at testing and baseline (enrollment) values as covariates. The means shown were adjusted for these covariates. Note the substantial Effect sizes for both physiological measures, indicating effects of MCEP may be observable in a small classroom size sample.

With regard to the primary outcome variables, HR was lower and vagal tone was higher in the MCEP group. (See Figure 3).

Analyses of covariance testing for group differences at the 6-month time point were also run for SDNN, RMSSD, low frequency R-R interval variability (LF), high frequency R-R interval variability (HF) (see Table 4). Three of these measures of HRV: SDNN (*p*-value = 0.027); RMSSD (*p*-value = 0.047) and LF power (*p*-value = 0.028) were significantly higher in the MCEP group. The sixth variable, HF power, did not quite reach statistical significance (*p*-value = 0.073). Effect size calculations also showed that all HRV parameters assessed were much greater in children who underwent the intervention than those who did not (R-R interval Hedge’s g = 1.09, SDNN Hedge’s g = 1.00, RMSSD Hedge’s g = 0.89, LF-power Hedge’s g = 0.98, HF-power Hedge’s g = 0.80, RSA Hedge’s g = 0.91).

Hypothesis 2. Vagal tone and HR would be correlated (positively and negatively respectively) with scores on the WECS (i.e., higher WECS scores would be associated with higher vagal tone and lower HR during the 20-min recovery period.

The test of Hypothesis 1 showed that children in the intervention group had lower heart rates and higher vagal tone at the six-month follow-up. The following analyses tested the hypothesis that at this time point there would be significant relationships (correlations) between WECS scores and physiology. Results showed that for the Attraction domain and for the total WECS scores there were significant negative correlations with HR; the children of dyads showing greater mutual attraction and total WECS scores had lower HRs. In addition, for Attraction there was significant positive correlation with RSA. These results are given in Table 3.

**Table 3:** Correlations between WECS scores and HR and RSA at the 6-month follow-up.

WECS Domain	HR	RSA
Attraction	**(24) *r* = −0.511, *p* = 0.011** *	**(24) *r* = 0.492, *p* = 0.015** *
Vocal	(22) *r* = −0.367, *p* = 0.092	(22) *r* = 0.270, *p* = 0.225
Facial	(23) *r* = -.357, *p* = 0.094	(23) *r* = 0.267, *p* = 0.218
Sensitivity	(24) *r* = -.380, *p* = 0.067	(24) *r* = 0.313, *p* = 0.136
Total	**(24) *r* = -.407, *p* = 0.048** *	(24) *r* = 0.053, *p* = 0.091

Data are for the 24 subjects with good physiological recordings at baseline and 6-month included in the results above. Results are for the mutual domain and total WECS scores. Some subjects had missing WECS codes for some domains (*n*).

Bolded numbers indicate significant *p*-value.

* Indicates that *p*-values remained <0.05 after controlling for child age, sex and group.

**Table 4:** Group differences in various measures of heart rate variability.

HRV parameter	FNI		SC		*P*-value	Effect size
	Mean	SD	Mean	SD		
R-R Interval (s)	0.59	0.04	0.55	0.04	**0.017**	**1.09**
SDNN (s)	0.05	0.01	0.04	0.01	**0.027**	**1.00**
RMSSD (s)	0.03	0.01	0.03	0.01	**0.047**	**0.89**
LF-power (see Table 2)	−6.21	0.51	−6.73	0.51	**0.028**	**0.98**
HF-power (see Table 2)	−7.50	0.71	−8.09	0.71	0.073	**0.80**
RSA (ln ms^2^)	4.54	0.72	3.86	0.72	**0.043**	**0.91**

FNI, family nurture intervention preschool; SC, standard curriculum; SD, pooled-weighted standard deviation.

Bolded *P*-value numbers = <0.05. Bolded Effect Size = highly significant.

Group Mean values were adjusted for baseline measurement, sex, and age at 6-months). Effect Size calculations are Hedge's *g* with a 95% CI.

Hypothesis 3. HR and vagal tone of children in the MCEP group (vs. control group) would be associated with better behavioral outcomes as assessed by the SWYC.

In a prior report we found that MCEP resulted in improvements in behavior as measured on the Survey of Well Being of Young Children (SWYC) (13). Here, we found children with worse SWYC scores tended to have higher heart rate (r = +0.39, *n* = 24, *p* = 0.057, effect size = 0.41).

## Discussion

The primary aim of this study was to assess the impact of MCEP on autonomic markers of socioemotional physiology in a preschool population. Despite the reduced number of subjects due to COVID, our findings are consistent with the interpretation that the MCEP intervention (vs. controls) had sustained effects on parasympathetic activity, including heart rate and vagal tone.

Consistent with our hypothesis, children in the MCEP group (vs. control group) showed significantly lower heart rates (longer R-R intervals) and increases in RSA and several measures of HRV at approximately 6-months following the intervention. In addition, the Hedge's g values for all HRV parameters for MCEP vs. controls results were quite large.

Analyses of the relationships between WECS emotional connection scores and physiology showed that children in dyads with the lowest WECS scores (i.e., less emotionally connected) had higher HRs and lower vagal tone (RSA) than children in the better-connected dyads. Moreover, we have previously reported there was an effect of MCEP on behavioral problems as measured by the SWYC (13). Fewer problems on the SWYC correlated with higher WECS scores (13), suggesting behaviors measured on the SWYC should also correlate with physiology. In fact, that is what we found. HR was correlated with SWYC behaviors. Taken together, these findings support a link between WECS behaviors and autonomic state.

### Theoretical and research considerations

Over the past few decades, vagal tone research among newborns and children has yielded important insights into social behavior, social interactions, and human psychology (28, 29). Heart rate variability, defined as the variation in intervals between consecutive heartbeats, has been used as a physiological indicator of regulatory processes, reflecting changes in autonomic regulation of heart rate. The sympathetic nervous system and parasympathetic nervous system both play a role in the regulation of heart rate, and to some extent can be indexed by measures of variability (30, 31). For example, the standard deviation of R-R peak intervals reflects overall variability, which is influenced by both sympathetic and parasympathetic nervous system activity (SNS and PNS respectively). Whereas, variation in successive R-R intervals, often termed high frequency HRV or respiratory sinus arrhythmia (RSA), largely reflects PNS activity mediated by the vagus nerve.

Vagal tone helps maintain the dynamic autonomic regulation important for cardiovascular health. In a healthy human heart, there is a dynamic relationship between the PNS and SNS. PNS control predominates at rest, resulting in an average (adult) HR of 75 bpm (36). The vagus nerve can exert its effects more rapidly (<1 s) than sympathetic nerves (>5 s) (37). Since these divisions can produce opposite actions on HR, their net effect on HR depends on their balance of activity. While the SNS often changes reciprocally to PNS activity, under some conditions the two can be activated or increased at the same time (i.e., co-activated) (38). While there is widespread agreement that modulation of heart rate variability or *vagal tone* helps maintain the dynamic autonomic regulation important for cardiovascular health, there are anomalies in the data, referred to as the *vagal paradox* (49) as well as in the possible evolutionary underpinnings of the relationship between vagal tone and socio-emotional regulation. We have proposed that this relationship can be explained by what we have termed the *autonomic socioemotional reflex (ASR*), which in this study is an external cardiac feedback loop mechanism dependent on mother-child co-regulation. This external loop is distinct from the internal self-regulating CNS feedback loop mechanisms.

We posit that the ASR (24) is present in all vertebrate species, and is arguably mediated by the oldest and most highly conserved learning mechanism—*functional Pavlovian or autonomic conditioning* (21, 25). Our theoretical advance is that the autonomic conditioning mechanism can be exploited (e.g., via calming cycle intervention) to lower average resting HR in the face of socioemotional challenge (26).

Previously, we tested aspects of the ASR and calming cycle theories (21, 22, 50) among prematurely born infants and their mothers in two RCTs of *Family Nurture Intervention* in the neonatal intensive care unit (FNI-NICU) (51). Results from both trials supported the study hypotheses. In the original trial, FNI led to greater maternal sensitivity during routine caregiving behavioral interactions (52), lowered infant heart rate in the hospital (26), enhanced autonomic regulation at term age (53), accelerated brain maturation in frontal regions at term age (54, 55), lowered symptoms of maternal depression at four months (27), and improved neurobehavioral outcomes at 18 months of age (56) and improved theory of mind at 4 and 5 years of age (57). In the two RCTs of FNI-NICU, EEG analyses showed that FNI mother-infant calming sessions in the NICU increased prefrontal power (44) and altered the development of brain-wide cortical activity networks such that by term age they closely resembled a comparison group of full-term infants (58).

The results of this preschool study suggest that a calming cycle intervention like that used in the FNI-NICU trials may be effective in regulating adverse socioemotional behavior in preschool aged children 2–5 years of age.

### Implications for practice

**Figure 4:**
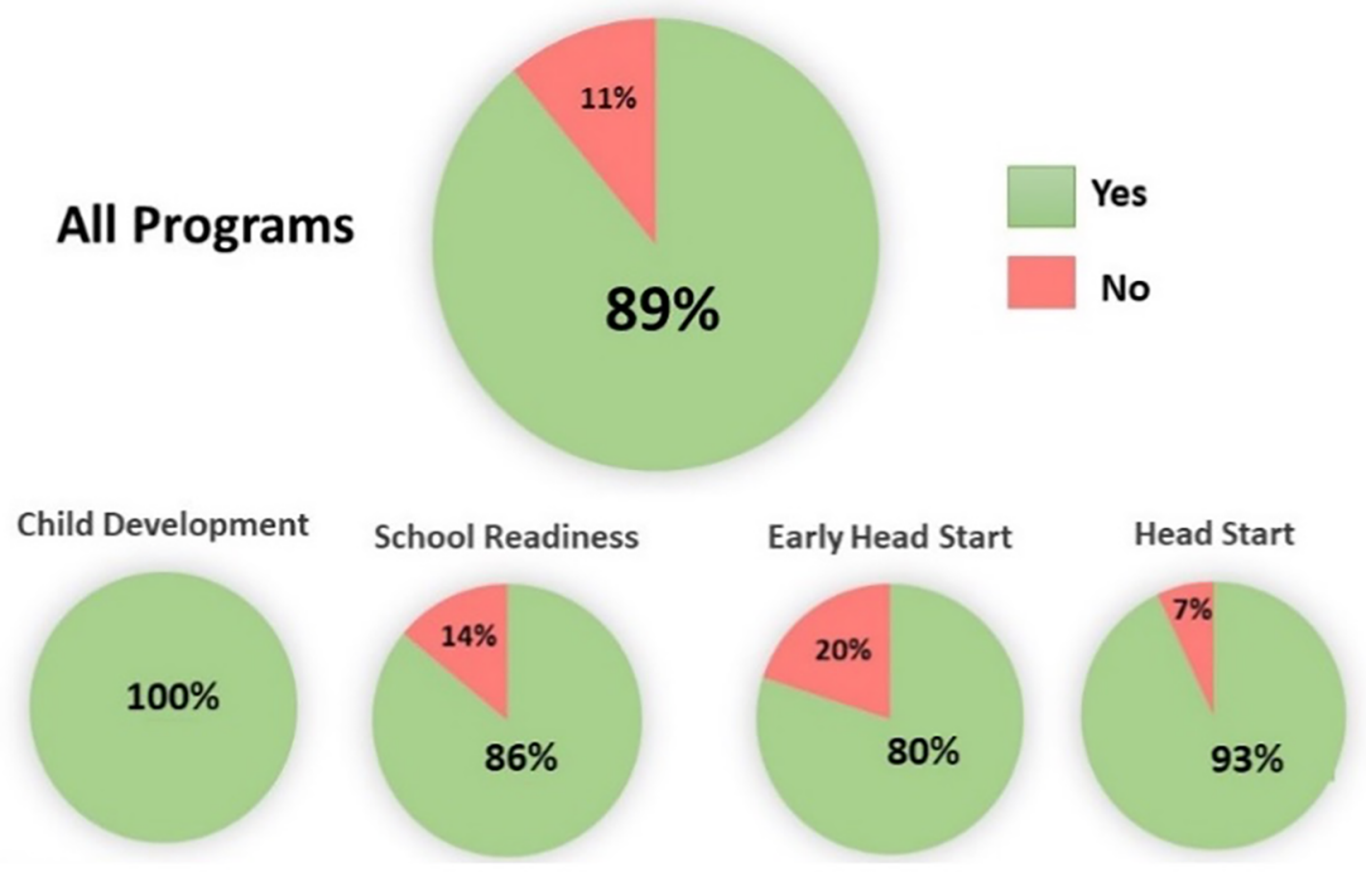
Percent staff who want to make MCEP part of standard CLC curriculum. Breakdown by CLC program.

Viewing the parent-child relationship as being mediated by the ASR has two distinct clinical advantages. First, as mentioned above, the primary reflex provides a novel target that avoids the negative stigmatizations associated with mental health. The MCEP intervention involves routines that can be practiced by the family in the home. Second, the ASR provides new ways to quickly assess the health of the parent-child emotional relationship (i.e., whether the relationship is adaptive or maladaptive). Since the ASR physiology is correlated with WECS behaviors, the behavioral state of the relationship can be monitored moment to moment with the WECS. The hypotheses and study design of this effectiveness trial were formulated based on CLC's routine standard preschool curriculum conditions. Therefore, the outcomes of the trial provided essential data necessary to inform possible changes in curriculum going forward. For instance, following completion of the study, based in part by observed changes in MCEP child behavior, CLC staff indicated they supported making MCEP part of standard CLC curriculum (See Figure 4). Teaches also indicated that MCEP participation helped strengthen the important parent-teacher alliance.

MCEP targets the parent-child relationship, the foundation of socioemotional behavior regulation (59). This approach is potentially less costly than comparable behavioral intervention models. MCEP is designed to reduce the child's stress reactivity by engaging parents in the child's autonomic nervous system conditioning at home. Current models, which are typically designed to change the thought processes of the parent or child and to help the child self-regulate emotions at school or in external programs, create a financial burden to the school and community (4).

Finally, the large effect size in our classroom-size sample is noteworthy (60). Robert Coe, from the School of Education, University of Durham, England explains why (61). “Effect size’ is a simple a way of quantifying the size of the difference between two groups. It is easy to calculate, readily understood and can be applied to any measured outcome in Education or Social Science. It is particularly valuable for quantifying and comparing the effectiveness of a particular intervention.” A strength of our study is that the results allow CLC to move beyond, “Does it work or not?” to the far more useful, “How well does it work in the standard classroom?” Not only was the efficacy of the MCEP statistically significant, but the size of the effect was also large, meaning that MCEP can have a positive effect on child behavior within the average preschool classroom. In many ways, therefore, the size of the effect might be the most important result of our study.

### Construct validity

The *emotional connection* construct describes a shared behavioral state, which is measurable via the WECS. Our previously published behavioral findings (13) validate the emotional connection construct in a preschool-aged child population. The construct c*o-regulation* describes shared autonomic state physiology and points to an external feed-back loop control system, in this case between mother and child (22). This is in stark contrast with current theory, which holds that emotional behavior is subject to an internal feed-back loop control system. The fact that emotional connection correlates with the child's autonomic state, and the child's autonomic state can be changed through the calming cycle intervention strongly suggests that the parent, the mother in this case, is key in the regulation of both the child's autonomic state and the child's behavior. We have proposed a sensory signaling pathway by which this co-regulation occurs (22).

It is noteworthy that the *baseline* EC state of the dyad was associated with recovery from stress. This finding is consistent with our work with infants (23) and extends it to young children, with further evidence that higher EC is associated with the child's ability to rejoin mother following relational stress and recover in a healthy autonomic state. Relational stress, such as waiting for teacher attention in the preschool classroom, is commonplace. Children who can manage such daily stressors with a physiological state of calmness may be less likely to show anger, impatience, and other forms of dysregulation that place them at-risk for removal from the preschool classroom.

There are additional aspects to this study and its findings that should also be noted. As stated in the introduction, there have been other intervention programs that address emotional, behavioral, and developmental deficits in children. MCEP differs from these programs in its simplicity and brevity, and ability to produce the physiological results seen in this study with eight or fewer 2-hour group sessions. In addition, the effects we see among children who received the intervention as compared to those who did not were measured at the 6-month timepoint, approximately 4 months after the intervention program was finished, indicating the intervention's sustained effect over time.

### Limitations

Several limitations to this study should be considered. Firstly, this study was done by recruiting a non-probability convenience sample of mainly low socioeconomic status and Hispanic-identifying individuals. The most significant limitation of the study was the small sample sizes for subjects with both baseline and 6-month data. Due to the impact of the COVID-19 pandemic, acquisition of physiological data was halted because a sizeable portion of the study's original sample were not able to come in for their 6-month timepoint physiology assessment. Comparing the demographic data of those enrolled vs. those who returned, the only significant difference observed between the two samples was that the original sample had a greater proportion of males in the intervention group compared the intervention group of this study. Yet, such a small sample size, it could be possible that these results are due to chance. However, the large effect sizes found in the study support the efficacy of the intervention.

### Future studies

Additional analyses are needed to explore several more questions. For example, it would be interesting to see if the change we report here in 6-month heart rate variability occurs gradually or suddenly. It would also be interesting to include other variables into the model that account for variation between subjects. Lastly, with a larger sample it would be interesting to see if covariates such as race, ethnicity and socioeconomic status influence these findings.

## Data Availability

The raw data supporting the conclusions of this article will be made available by the authors, without undue reservation.
